# High-intensity resistance training in patients with myositis – 1-year follow-up on a randomised controlled trial

**DOI:** 10.1007/s00296-025-05858-8

**Published:** 2025-04-16

**Authors:** Kasper Yde Jensen, Per Aagaard, Charlotte Suetta, Jakob Lindberg Nielsen, Rune Dueholm Bech, Henrik Daa Schrøder, Jan Christensen, Casper Simonsen, Louise Pyndt Diederichsen

**Affiliations:** 1 Copenhagen Research Center for Autoimmune Connective Tissue Diseases (COPEACT), Center for Rheumatology and Spine Diseases, Rigshospitalet, Copenhagen, Denmark; 2 Department of Sports Science and Clinical Biomechanics, University of Southern Denmark, Odense, Denmark; 3 Geriatric Research Unit, Department of Geriatric and Palliative Medicine, Copenhagen University Hospital, Bispebjerg and Frederiksberg, Copenhagen, Denmark; 4 Department of Orthopaedics and Traumatology, Zealand University Hospital, Koege, Denmark; 5 Department of Pathology, Odense University Hospital, Odense, Denmark; 6 Department of Occupational Therapy and Physiotherapy, Copenhagen University Hospital - Rigshospitalet, Copenhagen, Denmark; 7 Centre for Physical Activity Research, Copenhagen University Hospital - Rigshospitalet, Copenhagen, Denmark; 8 Department of Rheumatology, Odense University Hospital, Odense, Denmark; 9 Department of Clinical Medicine, Faculty of Health and Health Sciences, University of Copenhagen, Copenhagen, Denmark; 10 Copenhagen Center for Arthritis Research, Center for Rheumatology and Spine Diseases, Centre for Head and Orthopaedics, Copenhagen University Hospital-Rigshospitalet Glostrup, Copenhagen, Denmark

**Keywords:** Strength training, non-pharmacological treatment, long-term exercise effects

## Abstract

Reduced quality of life in patients with myositis is partly due to impairments in muscle strength, muscle endurance and functional capacity. In a recent randomised controlled trial (RCT) (NCT04486261) we showed that high-intensity resistance training improved quality of life, muscle strength, and endurance. This follow-up study aimed to investigate if these improvements remained persistent one year after completing 16 weeks of high-intensity resistance training. A total of 32 participants (intervention group (IG): 15; control group (CG): 17) were enrolled in the RCT, with 27 (IG: 13; CG: 14) completing the 1-year follow-up. Outcomes were assessed at three time points: baseline (weeks − 4 − 0), post-intervention (weeks 17–18), and 1-year follow-up (weeks 52–54). Outcomes included quality of life (QoL, SF36), functional capacity, muscle endurance (functional index 3 (FI3)), body composition (DEXA), and disease activity/damage, including manual muscle test 8 (MMT8). Training-induced improvements in muscle endurance (FI3) remained at 1-year follow-up, with a mean change of 10.7 (CI95: 2.2;19.1) in favour of IG (*p* = 0.01). Within-group improvements in IG were sustained for QoL (SF36, physical component summary) (4.8 (CI95: 0.9;8.7), *p* = 0.02), muscle strength (MMT8) (1.8 (CI95: 0.8; 2.9), *p* < 0.01), and functional capacity measures (*p* ≤ 0.04), although not significantly different from CG at 1-year follow-up. Disease activity and disease damage were similar between IG and CG at 1-year follow-up. Patients with myositis completing 16 weeks of high-intensity resistance training showed sustained improvements in muscle endurance at 1-year follow-up compared to controls. Indications of lasting enhancements in quality of life and strength were also present, with no increase in disease activity or damage. NCT04486261.

**Supplementary Information:**

The online version contains supplementary material available at 10.1007/s00296-025-05858-8.

## Introduction

Idiopathic inflammatory myopathy, commonly known as myositis, refers to a heterogeneous group of rare, autoimmune muscle diseases characterized by muscle inflammation. The clinical features are muscle weakness, decreased muscle endurance, and impaired physical function [1, 2]. In addition to the primary muscle involvement, patients often experience complications affecting the lung, heart, joints and skin [3, 4]. Immunosuppressive treatment is commonly employed to achieve disease remission in patients with myositis [3–5]. Yet, muscle weakness and reduced functional capacity often persist despite remission [6, 7] and these persistent symptoms have been shown to negatively impact the patients’ quality of life (QoL) [6–10]. Consequently, patients with myositis have impaired QoL compared with healthy age-matched individuals [6, 10–13], and interventions that can prevent loss of muscular function and in turn improve QoL are warranted.

To this end, we initiated a randomised controlled trial (RCT) to investigate the effect of high-intensity resistance training in patients with myositis [14]. In response to 16 weeks of resistance training, we observed significant and clinically relevant improvements in quality of life, muscle endurance and muscle strength. Importantly, this was achieved without affecting disease activity or disease damage [14]. These findings align with prior reports [15–20], however, to what extent these effects are sustained following cessation of the intervention remains unclear. To our knowledge, only three prior studies have included data on long-term follow-up [19–21]. All three studies reported sustained improvements in specific aspects of QoL and functional capacity when assessed 8–19 months following the cessation of training [19–21].

Against this background, the present study aimed to investigate if the engagement in a 16-week high-intensity resistance training intervention resulted in sustained improvements in QoL and functional performance at 1-year follow-up in adults with established myositis.

## Methods

### Study design

**Fig. 1:**
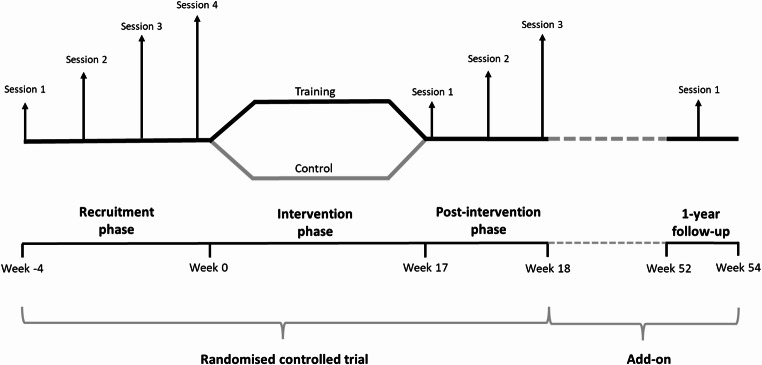
Study design

The present study was designed as a single-centre 1-year follow-up analysis of a two-armed, single-blinded RCT [14] to test the superiority of high-intensity resistance training compared with usual care to induce persistent gains in mechanical muscle function and functional capacity (Fig. 1). The original study was designed following the SPIRIT guidelines [22]. Details about the study design and primary results have been published previously [14, 23]. Briefly, the RCT was conducted at Copenhagen University Hospital – Rigshospitalet, Denmark, where participants were randomized 1:1 to an intervention group (IG) that performed 16 weeks of high-intensity resistance training in addition to usual care, or a control group (CG) receiving usual care only including immunosuppressives [14].

The study was approved by The Danish National Committee on Health Research Ethics (H-20030409) and The Danish Data Protection Agency (P-2020–553), while prospectively registered at ClinicalTrials.gov (NCT04486261). All experimental procedures were conducted in accordance with the Declaration of Helsinki. All study participants provided their written informed consent before engaging in the study.

### Participants

Between February 2021 and May 2021, 160 patients aged ≥ 18 years fulfilling the criteria for idiopathic inflammatory myopathies by EULAR/ACR [24, 25] and affiliated with a tertiary care centre for myositis in the Capital Region of Denmark, were screened for eligibility.

As described previously [14], the specific inclusion criteria were a diagnosis of myositis ≥ 6 months, ≤ 5 mg of oral prednisolone daily and stable immunosuppressive treatment ≥ 1 month before entering the study. The exclusion criteria were having inclusion body myositis, additional systemic autoimmune disease (except Sjögrens Syndrome) or comorbidity preventing resistance training (i.e., severe heart/lung disease, uncontrolled hypertension, severe knee/hip arthritis).

Adjustments in dose or change of immunosuppressive treatment were not permitted during the intervention period study and would cause termination of participation in the study.

### Randomisation and blinding

Participants were randomly allocated to IG or CG using randomization software (Sealed Envelope Ltd. 2021) with computer-generated allocation sequences [14]. The allocation sequences were generated using permuted blocks and were stratified for age (2 levels: <50 years or ≥ 50 years) and lung involvement (2 levels: Yes or No). Allocation was performed following the last day of baseline testing by the principal investigator (KYJ), allowing the blinding of outcome assessors. The randomization software ensured allocation concealment and only the principal investigator (KYJ) had access to the allocation sequence.

### Intervention procedures

#### High-intensity resistance training

As described in detail elsewhere [14], participants in IG were allocated to perform supervised high-intensity resistance training twice a week for 16 weeks, in addition to receiving usual care. The duration of an exercise session was ∼1 h and started with a 10-minute warm-up on a stationary ergometer bike at a low-to-moderate workload (25–100 Watts). Subsequently, a machine-based resistance exercise program consisting of horizontal bench press, horizontal leg press, seated rows, knee extension and seated biceps curls was performed. The training protocol comprised 3 sets of 10 repetitions for each exercise, with the training intensity set at 10 repetitions maximum (RM). The initial intensity was individualised based on a measurement of 5 RM strength at baseline [26]. Throughout the 16 weeks of training, the exercise loads (kg) were progressively adjusted. The load was increased when a participant could perform three additional repetitions in the third and final set of the respective exercise. The first four training sessions served as familiarisation to the training regime, using lower loading intensities of 15RM performed in 10 repetitions for all exercises.

#### Usual care

Control participants (CG) received usual care, which consisted of continuing the current treatment regimen (incl. immunosuppressant treatment) and participating in myositis-related visits at the hospital, per usual. Further CG were instructed to maintain their habitual physical activity levels throughout the intervention period. No study-related contacts (i.e., phone calls, emails, and visits) were taken during the intervention period.

#### Post-intervention exercise recommendations

Participants in both IG and CG were encouraged to perform weekly exercise sessions following the 4-month intervention period. All participants were recommended to follow an exercise protocol using unsupervised home- or gym-based exercises. The optional Gym-based training protocol involved two weekly exercise sessions with a full-body protocol of 7 different exercises. The volume of the respective exercise was set at three sets of 10 repetitions and the intensity was set at 10 RM. Participants were encouraged to progress to higher loads (kg) when they were able to perform three additional repetitions in the last (i.e., third) set of the respective exercise (suppl. Table A). Participants in CG were offered a supervised training session in which the training protocol was explained, all exercises were demonstrated and subsequently performed by the participant.

All participants received elastic (resistance) bands (loads equivalent to 10–60 kg) enabling exercise sessions at home and were asked to complete a training dairy (suppl. Figure A).

Two months following the end of the intervention (i.e., six months following baseline), a meeting was held for all participants and their families to share the results from the intervention study. No other study-related contacts occurred between post-intervention and 1-year follow-up.

#### Outcome assessments

Patient demographics and disease characteristics were extracted from the electronic medical records at baseline. All outcome measures (excl. muscle biopsies and accelerometer data) were obtained at baseline, post-intervention and 1-year follow-up. The lead physician (LPD) assessed all physician-related outcomes at all time points, while DEXA scans and physical performance tests (excluding functional index 3 – FI3) were performed by two trained physiotherapists blinded to group allocation. The principal investigator (KYJ) performed FI3 testing at all time points.

A detailed description of the assessment procedures for all outcome variables obtained in relation to the RCT (NCT04486261) has been presented previously [14, 27].

#### Quality of life

Quality of life (QoL) was assessed using the Short Form-36 health questionnaire v.1 (SF36) [28]. The Physical Component Summary (PCS) consists of four subscales (physical functioning, pain, general health, and physical roles). Likewise, the Mental Component Summary (MCS) consists of four subscales (vitality, social functioning, emotional roles, and mental health) [29].

#### Functional capacity

Functional Index 3 (FI3) measures muscle endurance in myositis patients [30] through shoulder, neck, and hip flexion tasks on both sides, paced by a metronome (40 beats/minute). Participants did five practice reps, then as many as possible up to 60. The 2-minute walk test (2MWT) assessed gait and short-term endurance [31] on a 20-meter track, aiming for the longest distance in two minutes. The Timed Up & Go (TUG) test measured functional mobility [32], where participants stood from a chair, walked three meters, turned, walked back, and sat down quickly. The fastest of three attempts was used. The 30-second sit-to-stand (30-STS) test measured lower body strength [33], with participants standing and sitting as many times as possible in 30 s. Postural balance was tested using the Short Physical Performance Battery [34], involving three 10-second stances (feet together, semi-tandem, full tandem) without arm support. Scores ranged from 0 to 30 s, with higher scores indicating better balance [34].

#### Unilateral leg extensor muscle power

The assessment of muscle power was conducted in the Nottingham power rig [28]. Participants performed a minimum of five times with 15–20 s of rest between the attempts. Participants were seated to ensure a knee joint angle of 15 degrees (with 0 degrees representing full extension) when the footplate was fully extended [28]. The trial with the relative highest peak power (watts/kg bodyweight) was selected for further analysis.

#### Handgrip strength

A static hand dynamometer (Jamar, JLW Instruments, USA) was used to assess maximal handgrip strength. Participants performed three trials with their dominant hand, with a 60-second rest period between each trial. The participants were seated upright with the arm bent at 90° at the elbow and were not allowed to use their legs to support the arm [29]. The trial with the highest peak force was selected for analysis.

#### Body composition

DEXA (Whole body) scans were performed with an iDXA fan-beam densitometer (GE Lunar, Madison, Wisconsin, USA) and used the Encore software (v. 16.0) for analyses. The body composition scans were performed before the physical testing. Data extracted for the current study consisted of weight (kg), BMI (kg/m^2^), total lean mass (kg), total fat mass (kg), relative fat mass (kg/m^2^) and adjusted appendicular lean mass, which was calculated as the sum of arms and legs, adjusted for height [30].

#### Activity levels

The International Physical Activity Questionnaire - Danish (IPAQ) assessed self-reported physical activity, calculating active and sedentary hours per week [31].

#### Disease-related outcomes

The International Myositis Assessment and Clinical Studies Group (IMACS) core set measures quantified disease activity and damage [32]. Evaluations included physician (PhGA) and patient global activity (PtGA), extramuscular global assessment (EMGA), manual muscle testing (MMT8), health assessment questionnaire (HAQ), and plasma creatine kinase (CK) levels. Disease damage was assessed using physician (PhGD) and patient global damage (PtGD) scores [32].

### Statistical considerations

#### Sample size

The initial RCT study was designed as a superiority trial, that aimed for a 20% change in the primary endpoint, the SF36 physical component summary. The power for the trial was set at 80%, with a statistical significance level of 0.05 (two-tailed) and a 10% dropout rate, leading to an estimated number of 60 patients [23].

#### Statistical analysis

Statistical analyses were conducted to examine the change in specific outcome variables from baseline to 1-year follow-up and from post-intervention to 1-year follow-up, respectively. All statistical analyses were conducted according to the “intention-to-treat” principle [33]. Continuous outcome variables were analyzed using constrained longitudinal data analyses via linear mixed models (LMMs) [34]. The LMMs contained fixed effects of time (3 levels: baseline, post-intervention, and 1-year follow-up), treatment (coded 0 for both groups at baseline and 0, 1 for CG and IG on the other time points), time x treatment interaction, and a random intercept for participant ID. The estimated between-group differences in change are reported with 95% confidence intervals. Similarly, within-group changes were calculated as changes from baseline to 1-year follow-up and from post-intervention to 1-year follow-up with 95% confidence intervals. No covariates were included in the LMM analysis. Based on the previous analyses of correlations [27],, we performed adjusted analyses in which fat percentage was included in the models as fat percentage had the highest negative correlation with QoL (suppl. Table B-D). Statistical significance was set at *p* ≤ 0.05 using two-tailed testing.

All values are presented as group means (with standard deviation) unless otherwise stated. R Studio (V. 2024.04.2 + 764) was used for all statistical procedures.

## Results

### Baseline demographics and clinical characteristics

Recruitment took place from February to May 2021 but concluded before reaching the target of 60 patients due to a lack of additional eligible patients with myositis in the Capital Region of Copenhagen. The post-intervention phase occurred from June to September 2021, and the 1-year follow-up was conducted from February to May 2022. As previously described [14], 32 patients with myositis were randomly allocated to the intervention group (IG) (*n* = 15) or the control group (CG) (*n* = 17). Baseline demographics and clinical characteristics are shown in Table 1.

**Table 1:** Baseline demographics, clinical characteristics, and core set measures of participating patients with myositis

	IG (*n* = 15)	CG (*n* = 17)	Total (*n* = 32)
Female, *n (%)*	10 (66.7%)	11 (68.7%)	21 (65.6%)
Age, *Years*	44.9 ± 18.9	50.3 ± 14.7	48.8 ± 16.7
Caucasian, *n* (%)	15 (100%)	16 (94%)	31 (96.9%)
Disease duration, Y*ears*	5.8 ± 4.7	4.4 ± 3.5	4.8 ± 4.1
IIM subset, *n (%)*^*+*^			
* Dermatomyositis*	3 (20.0%)	5 (29.4%)	8 (25.0%)
* Amyopathic dermatomyositis*	1 (6.7%)	1 (5.9%)	2 (6.3%)
* Juvenile dermatomyositis*	4 (26.7%)	1 (5.9%)	5 (15.6%)
* Antisynthetase syndrome*	5 (33.3%)	7 (41.2%)	12 (37.5%)
* Immune-mediated necrotizing myopathy*	1 (6.7%)	2 (11.8%)	3 (9.4%)
* Unspecified myositis*	1 (6.7%)	1 (5.9%)	2 (6.3%)
Myositis-specific autoantibodies, *n* (%)			
* Anti-Jo-1*	4 (26.7%)	7 (41.2%)	11 (34.4%)
* Anti-OJ*	1 (6.7%)	0 (0%)	1 (3.1%)
* Anti-SRP*	1 (6.7%)	0 (0%)	1 (3.1%)
* Anti-HMGCR*	0 (0%)	2 (11.8%)	2 (6.3%)
* Anti-Mi2*	0 (0%)	1 (5.9%)	1 (3.1%)
* Anti-NXP2*	2 (13.3%)	1 (5.9%)	3 (9.4%)
* Anti-MDA-5*	1 (6.7%)	1 (5.9%)	2 (6.3%)
* Anti-TIF1-γ*	1 (6.7%)	2 (11.8%)	3 (9.4%)
Myositis-associated autoantibodies, *n* (%)			
* Anti-Ro*	1 (26.7%)	6 (35.3%)	7 (21.9%)
* Anti-Pm/Scl-75*	2 (13.3%)	1 (5.9%)	3 (9.4%)
* Anti-Pm/Scl-100*	1 (6.7%)	1 (5.9%)	2 (6.3%)
Extramuscular organ involvement^+^			
* Interstitial lung disease*,* n (%)*	8 (53.3%)	10 (58.8%)	18 (56.3%)
* Arthritis*,* n (%)*	8 (53.3%)	10 (58.8%)	18 (56.3%)
* Raynaud*,* n (%)*	7 (46.7%)	5 (29.4%)	12 (37.5%)
* Dysphagia*,* n (%)*	9 (60.0%)	8 (47.1%)	17 (53.1%)
Disease activity measures			
* Creatine kinase*,* mmol/L* * Reference value: 40–280 mmol/L*	277 ± 460	162 ± 172	216 ± 338
* Health assessment questionnaire*,* 0–3*	0.21 ± 0.29	0.29 ± 0.61	0.25 ± 0.48
* Manuel muscle testing 8*,* 0–80*	76.7 ± 3.9	76.9 ± 2.6	76.8 ± 3.2
* Physician global activity*,* VAS 0–100 mm*	7.9 ± 8.8	6.6 ± 6.5	7.2 ± 7.6
* Patient global activity*,* VAS 0–100 mm*	4.3 ± 5.0	3.2 ± 3.5	3.8 ± 4.4
* Extramuscular global assessment*,* VAS 0–100 mm*	4.0 ± 5.4	3.2 ± 3.5	3.6 ± 1.5
Disease damage measures			
* Physician global damage*,* VAS 0–100 mm*	17.0 ± 13.2	15.0 ± 9.4	15.9 ± 11.2
* Patient global damage*,* VAS 0–100 mm*	19.7 ± 18.9	18.2 ± 14.1	18.9 ± 16.3
Immunosuppressives, *n (%)*	10 (66.7%)	15 (82.4%)	25 (78.1%)
* Prednisolone*	3^	2*	5
* sDMARD*	10	13	23
* bDMARD*	3^#^	3^¤^	6

Data are presented as mean ± SD or number & (%). IG: Intervention group trained with high-intensity resistance training; CG: Control group; sDMARD: Synthetic disease-modifying antirheumatic drugs; bDMARD: Biological disease-modifying antirheumatic drugs. ^all patients who received prednisolone also received sDMARD. ^#^ All patients who received bDMARD also received sDMARD, while two out of three also received prednisolone *One patient who received prednisolone also received sDMARD, while the other received bDMARD. ^¤^ One patient received bDMARD only

**Fig. 2:**
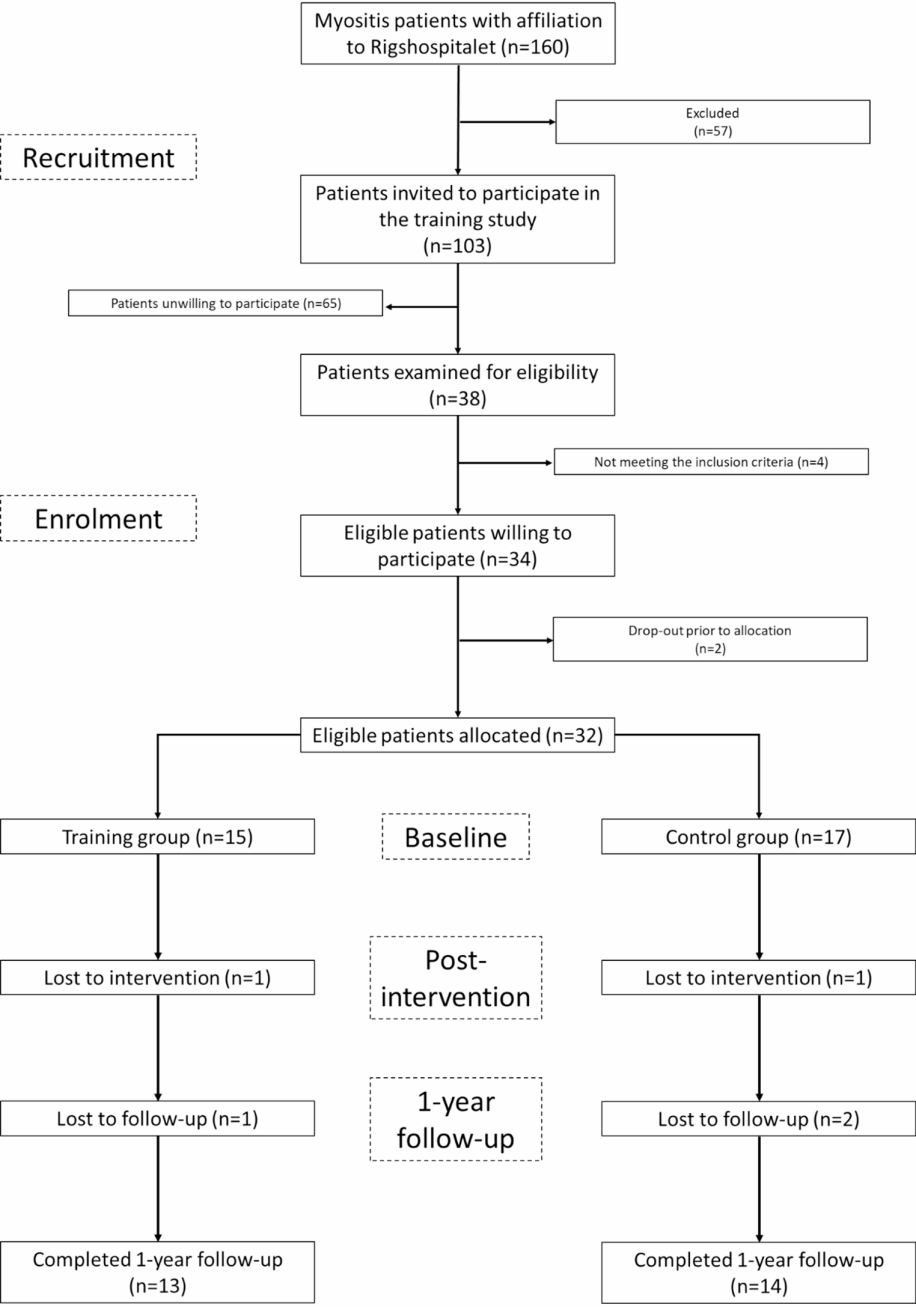
Patients’ flowchart

During the intervention period, two patients were lost to post-intervention (IG, *n* = 1; CG, *n* = 1). The IG patient dropped out of the study due to fatigue and the CG patient was excluded due to changes in medication (Fig. 2).

At 1-year follow-up, three additional patients were lost to follow-up (IG, *n* = 1; CG, *n* = 2). The IG patient found it too time-consuming to perform the 1-year follow-up visit, while the CG patients had non-training related injuries, which made measuring outcomes unreliable and was therefore excluded (Fig. 2). Only a single participant had filled out the training diary at the 1-year follow-up, thus making it impossible to extrapolate data on training patterns from post-intervention to 1-year follow-up.

#### Quality of life

The primary outcome from the initial RCT, the SF36 PCS, revealed a trend towards a between-group difference at the 1-year follow-up of 4.9 points [-0.2; 10.1] (*p* = 0.06) (Table 2). A within-group difference from baseline to 1-year follow-up 4.8 points [0.9; 8.7] (*p* = 0.02), was observed in IG with no changes from post-intervention to 1-year follow-up. In contrast, no changes from baseline or post-intervention to 1-year follow-up were observed in the CG (Table 2). No between-group differences in the SF36 MCS were observed at 1-year follow-up -6.9 points [-16.3; 2.5] (*p* = 0.15) (Table 2).

**Table 2:** Differences in quality of life, functional capacity and muscle power at 1-year follow-up

	Intervention (*N* = 15)				Control (*N* = 17)				Between-group difference	
	Baseline to 1 year		Post to 1 year		Baseline to 1 year		Post to 1 year		1-year follow-up	
	*Difference* *(95% CI)*	*P-value*	*Difference* *(95% CI)*	*P-value*	*Difference* *(95% CI)*	*P-value*	*Difference* *(95% CI)*	*P-value*	*Difference* *(95% CI)*	*P-value*
QoL – PCS summary score	*4.8* *(0.9; 8.7)*	*0.02*	*-0.2* *(-4.2; 3.9)*	*0.94*	-0.1 (-3.7; 3.5)	0.96	-0.8 (-4.6; 2.9)	*0.67*	4.9 (-0.2; 10.1)	0.06
QoL – MCS summary score	-0.4 (-7.9; 7.1)	0.92	-5.4 (-13.7; 2.8)	0.19	6.5 (-0.5; 13.6)	0.07	3.5 (-4.1; 11.2)	0.36	-6.9 (-16.3; 2.5)	0.15
FI3 (%)	10.2 (4.1; 16.3)	< 0.01	-6.5 (-12.7; -0.3)	0.04	-0.5 (-6.4; 5.4)	0.87	-6.1 (-12.1; -0.2)	0.04	10.7 (2.2; 19.1)	0.01
30-s STS (rep)	3.2 (1.6; 4.7)	< 0.01	2.1 (0.5; 3.6)	0.01	3.7 (2.2;5.2)	< 0.01	3.7 (2.2; 5.2)	< 0.01	-0.5 (-2.6; 1.6)	0.61
TUG (s)	-0.7 (-1.2; -0.2)	< 0.01	0.0 (-0.5; 0.5)	0.89	-0.5 (-0.9; 0.2)	0.04	-0.2 (-0.7; 0.2)	0.30	-0.2 (-0.9; 0.4)	0.50
2MWT (m)	6.8 (0.2; 13.4)	0.04	-1.5 (-8.2; 5.1)	0.65	11.9 (5.6; 18.2)	< 0.01	3.0 (-3.3; 9.3)	0.35	-5.1 (-14.1; 4.0)	0.26
Balance (s)	0.6 (-0.2; 1.4)	0.16	0.2 (-0.7; 1.1)	0.68	0.8 (0.0; 1.6)	0.06	0.4 (-0.5; 1.3)	0.42	-0.2 (-1.2; 0.9)	0.72
Handgrip (kg)	2.6 (-0.3; 5.5)	0.08	1.2 (-1.7; 4.2)	0.41	-0.8 (-3.6; 2.0)	0.58	-2.5 (-5.3; 0.3)	0.08	3.4 (-0.6; 7.4)	0.09
LEP (Watts/kg)	0.2 (-0.1; 0.5)	0.12	0.0 (-0.3; 0.3)	0.96	0.2 (-0.1; 0.4)	0.15	0.0 (-0.2; 0.3)	0.92	0.0 (-0.3; 0.4)	0.88

QoL: Quality of life, PCS: Physical component summary, MCS: Mental component summary, FI3: Functional index, 30-s STS: 30 s sit to stand, TUG: Timed Up & Go, 2MWT: 2-minute walk test, LEP: Leg extensor power

#### Functional capacity, postural balance, muscle power and strength

Functional index 3 (FI3) showed between-group differences at the 1-year follow-up of 10.7 points [2.2; 19.1] points (*p* = 0.01) (Table 2). IG demonstrated a within-group increase from baseline to 1-year follow-up of 10.2 points [4.1; 16.3] (*p* < 0.01). However, a decrease from post-intervention to 1-year follow-up of -6.5 points [-12.7; -0.3] (*p* = 0.04) was also noted. No changes from baseline to 1-year follow-up were observed in CG, while it decreased by 6.1 points [-12.1; -0.2] (*p* = 0.04) from post-intervention to a decrease from post to 1-year follow-up (Table 2). No between-group differences were found in 30-s STS performance, TUG, and 2MWT (Table 2). However, within-group improvements in IG from baseline to 1-year follow-up were observed for 30-s STS (3.2 points [1.6; 4.7] (*p* < 0.01)), TUG (-0.7 points [-1.2; -0.2] (*p* < 0.01)) and 2MWT (6.8 points [0.2; 13.4] (*p* = 0.04)). Likewise, in CG, within-group improvements from baseline to 1-year follow-up 30-s STS (3.7 points [2.2; 5.2] (*p* < 0.01)), TUG (-0.7 points [-1.2; -0.2] (*p* = 0.04)) and 2MWT (6.8 points [0.2; 13.4] (*p* < 0.01)) were observed (Table 2). For postural balance, handgrip strength and maximal unilateral leg extensor power, there were no between-group differences at 1-year follow-up (Table 2). No within-group decreases from post-intervention to 1-year follow-up were observed for any outcomes other than FI3, in both IG and CG (Table 2).

### Disease activity and damage

#### Disease activity and disease damage

No between-group differences in PhGA and PtGA were observed at 1-year follow-up (Table 3). Additionally, no between-group differences in the clinical measure of muscle strength MMT8 were seen at the 1-year follow-up (Table 3). Similarly, no between-group differences were found in EMGA, HAQ and plasma CK at the 1-year follow-up (Table 3). No between-group differences in PhGD or PtGD were observed at the 1-year follow-up (Table 3).

**Table 3:** Differences in disease activity and disease damage at 1-year follow-up

	Intervention (*N* = 15)				Control (*N* = 17)				Between-group difference	
	Baseline to 1 year		Post to 1 year		Baseline to 1 year		Post to 1 year		1-year follow-up	
	*Difference* *(95% CI)*	*P-value*	*Difference* *(95% CI)*	*P-value*	*Difference* *(95% CI)*	*P-value*	*Difference* *(95% CI)*	*P-value*	*Difference* *(95% CI)*	*P-value*
PhGA (0-100)	3.1 (-0.5; 6.7)	0.09	2.6 (-1.3; 6.4)	0.19	2.8 (-0.6; 6.3)	0.11	2.3 (-1.3; 6.0)	0.20	0.3 (-4.4; 5.1)	0.90
PtGA (0-100)	-3.9 (-7.4; -0.4)	0.03	-3.0 (-6.7; 0.7)	0.11	-1.9 (-5.3; 1.5)	0.26	-1.7 (-5.3; 1.8)	0.33	-2.0 (-6.7; 2.8)	0.41
EMGA (0-100)	-0.9 (-3.5; 1.6)	0.46	-1.2 (-3.8; 1.5)	0.38	0.8 (-1.6; 3.3)	0.51	0.6 (-2.0; 3.1)	0.66	-1.7 (-5.1; 1.6)	0.31
MMT8 (0–80)	1.8 (0.8; 2.9)	< 0.01	0.0 (-1.1; 1.1)	0.96	1.2 (0.2; 2.2)	0.02	1.0 (0.0; 2.0)	0.06	0.6 (-0.8; 2.0)	0.39
HAQ (0–3)	0.0 (-0.2; 0.1)	0.76	0.1 (-0.1; 0.2)	0.30	-0.1 (-0.2; 0.1)	0.31	0.0 (-0.2; 0.1)	0.78	0.0 (-0.1; 0.2)	0.62
CK (mmol/L)	-77 (-243; 90)	0.36	5 (-184; 193)	0.96	-75 (-237; 86)	0.36	-98 (-278; 82)	0.28	-15 (-205; 202)	0.99
PhGD (0-100)	-5.5 (-11.7; 0.8)	0.09	3.8 (-2.8; 10.4)	0.25	-5.7 (-11.7; 0.4)	0.07	-0.8 (-7.1; 5.4)	0.79	0.2 (-8.1; 8.5)	0.97
PtGD (0-100)	-5.1 (-8.6; -1.7)	< 0.01	-1.0 (-4.5; 2.6)	0.59	-4.5 (-7.8; -1.2)	< 0.01	-3.1 (-6.5; 0.2)	0.07	-0.6 (-5.3; 4.1)	0.79

PhGA: Physician global activity, PtGA: Patient global activity, EMGA: Extramuscular global activity, MMT8, Manual muscle testing 8, HAQ: Health assessment questionnaire, CK: creatine kinase, PhGD: Physician global damage, PtGD: Patient global damage

#### Body composition and physical activity

No between-group differences were observed at the 1-year follow-up for total weight, body mass index, total muscle mass, appendicular muscle mass, total fat mass, appendicular fat mass, IPAQ active hours and IPAQ sitting hours (Table 4).

**Table 4:** Differences in body composition and physical activity at 1-year follow-up

	Intervention (N = 15)				Control (N = 17)				Between-group difference	
	Baseline to 1 year		Post to 1 year		Baseline to 1 year		Post to 1 year		1-year follow-up	
	*Difference (95% CI)*	*P-value*	*Difference (95% CI)*	*P-value*	*Difference (95% CI)*	*P-value*	*Difference (95% CI)*	*P-value*	*Difference (95% CI)*	*P-value*
Weight (kg)	0.6 (-1.3; 2.4)	0.56	1.4 (-0.5; 3.3)	0.14	-1.2 (-3.0; 0.6)	0.19	-0.7 (-2.5; 1.1)	0.46	1.7 (-0.9; 4.4)	0.19
BMI (kg/m^2^)	0.2 (-0.4; 0.8)	0.47	0.5 (-0.1; 1.1)	0.12	-0.4 (-1.0; 0.2)	0.16	-0.3 (-0.8; 0.3)	0.39	0.7 (-0.2; 1.5)	0.13
Total lean mass (kg)	0.0 (-0.7; 0.6)	0.89	0.1 (-0.5; 0.8)	0.70	0.0 (-0.6; 0.6)	0.98	-0.2 (-0.8; 0.5)	0.58	0.0 (-1.0; 0.9)	0.93
App. lean mass (kg/m^2^)	-0.1 (-0.3; 0.1)	0.50	0.0 (-0.2; 0.2)	0.90	-0.1 (-0.3; 0.1)	0.38	0.1 (-0.1; 0.4)	0.20	0.0 (-0.3; 0.3)	0.91
Total fat (%)	0.9 (-0.8; 2.5)	0.30	0.3 (-1.3; 1.9)	0.72	-1.0 (-2.5; 0.6)	0.23	-0.5 (-2.1; 1.0)	0.51	1.8 (-0.4; 4.0)	0.11
App. fat mass (kg/m^2^)	0.1 (-0.2; 0.4)	0.54	0.2 (-0.2; 0.5)	0.27	-0.3 (-0.6; 0.1)	0.11	-0.4 (-0.7; -0.1)	0.02	0.4 (-0.1; 0.8)	0.12
IPAQ (Active hours/week)	-6.2 (-16.6; 4.1)	0.23	-4.2 (-15.0; 6.6)	0.44	4.5 (-5.1; 14.2)	0.35	7.1 (-2.9; 17.2)	0.16	-10.8 (-24.4; 2.9)	0.12
IPAQ (Sitting hours/week)	8.1 (-5.2; 21.4)	0.23	-5.0 (-19.5; 9.5)	0.49	9.8 (-2.7; 22.4)	0.12	6.8 (-6.7; 20.3)	0.32	-1.8 (-18.6; 15.1)	0.84

BMI: Body mass index, App.: Appendicular, IPAQ: International physical activity questionnaire

#### Post hoc analysis

Post hoc analyses were performed with fat percentage as a covariate for the full sets of outcome variables, excluding measures of body composition. Here, we observed a between-group difference of 6.1 points [1.0; 11.3] (*p* = 0.02) in PCS in favour of the IG, from baseline to the 1-year follow-up. For the remaining outcomes, the results of the adjusted analyses were in line with unadjusted analyses (suppl. Table B-D).

## Discussion

As a follow-up study to our previous RCT, the main finding of the present study was that 16 weeks of high-intensity resistance training led to persistent improvements in muscle endurance (i.e., FI3) at the 1-year follow-up compared to a control group. Additionally, the intervention group showed indications of sustained improvements in quality of life compared to the control group at 1-year follow-up. Likewise, various measures of functional capacity and muscle strength also remained enhanced in the intervention group at 1-year follow-up. Importantly, these effects were achieved without affecting disease activity and disease damage throughout the study period (baseline to 1-year follow-up). Altogether, our findings highlight the potential of incorporating high-intensity resistance training as a part of the non-pharmacological treatment for patients with myositis.

As previously reported, we observed a post-training improvement of 12% in the PCS score of the SF36 questionnaire in the intervention group, an effect that was not present in the control group [14]. This improvement in PCS showed a trend towards being sustained at the 1-year follow-up, indicating that even short (months) periods of supervised high-intensity exercise can be effective in inducing lasting and clinically relevant changes in QoL. Munters et al. included [19] a 1-year follow-up in their endurance-based exercise RCT study involving 12 weeks of ergometer cycling (70% of VO_2_ max) three times a week, in patients with poly- and dermatomyositis. They found a sustained improvement for the intervention group in the General Health subdomain of SF36 at the 1-year follow-up [19]. Alexanderson et al. presented a 2-year follow-up on a 24-week resistive home exercise programme twice a week [17] and found a lasting improvement in the domain of Energy in the Nottingham Health profile at both 1- and 2-year follow-up [20]. Finally, Tiffreau et al. performed an exercise study with four weeks of standardised hospital-based rehabilitation followed by a personalised, home-based rehabilitation program for patients with poly- and dermatomyositis [21]. During the 1-year follow-up, they found that the intervention group had sustained higher scores of the SF36 subdomains of General Health, Role Physical and Pain compared to the control group [21].

In line with our earlier observation of no change in MCS following 16 weeks of resistance training in the present cohort of myositis patients [14], no changes were observed at 1-year follow-up in either subject group. Likewise, none of the previous exercise-based RCTs found any changes at 1- or 2-year follow-up in outcome measures linked to the mental part of quality of life [19–21].

We previously have reported improvements in both muscle endurance (FI3) and muscle strength (MMT8) in established myositis patients following exercise-based intervention protocols [14]. In the present analysis, improvement in muscle endurance/functional capacity was found to persist at 1-year follow-up for the intervention group, which still showed superior results compared to the control group. Munters et al. [19] found that muscle performance assessed as 5-RM strength in an isolated knee extension test remained elevated in the intervention group at 1-year follow-up, while there was no change in the control group [19]. Alexanderson et al. found similar results at 2-year follow-up for the exercise intervention group (15 min of resistive exercise followed by stretching twice a week), with sustained improvements both in muscle performance (FI1) and aerobic capacity (8-min submaximal treadmill test) compared to baseline, while remaining unchanged in their control group [20]. In contrast, Tiffreau et al. [21] reported no differences in knee flexor and extensor muscle strength (Isokinetic assessments) between their intervention group and controls at 1-year follow-up [21].

The present muscle strength improvements following the 16 weeks of high-intensity resistance training were maintained within our intervention group at 1-year follow-up; however, follow-up levels were not statistically superior to the control group. Notably, Tiffreau et al., using the Kendall manual muscle test [35], demonstrated higher scores at 1-year follow-up for the intervention group on the left side compared to the control group, whereas no group differences were observed for muscles on the right side [21].

The interpretation of the present MMT8 results at follow-up needs to be made with caution, as there is a high likelihood of a ceiling effect masking the true effect in both subject groups, given that a substantial number of participants were able to achieve maximum scores already at baseline (*n* = 9) as well as following 16 weeks intervention (*n* = 12). This raises questions about the sensitivity of MMT8 as a stand-alone measure of skeletal muscle strength in stable patients with myositis. To overcome this problem, several groups have reported different add-ons to counter the ceiling effect of MMT8 by the addition of Functional index [36], 30-second sit-to-stand [37, 38], 2-minute walk test [37, 39] and (hand-held) dynamometer tests [40, 41].

Physical activity and body composition did not differ across the intervention and the control group at 1-year follow-up, consistent with the results reported in the initial RCT study [14]. As the present study didn´t have focus on weight loss or increasing overall physical activity levels, these observations may not be surprising. Notably, in our former study on baseline predictors for QoL, a negative correlation was observed between fat mass and QoL [27]. Further, previous observations have shown that higher levels of physical activity correlate with better clinical outcomes such as MMT8, HAQ and PhGD [42–44]. Altogether, these observations suggest that promoting weight loss (if relevant) and increasing physical activity, could be beneficial, as a part of the non-pharmacological treatment for patients with myositis.

Disease activity and disease damage are essential markers for monitoring disease progression in myositis patients [32] and thus may be considered important outcome measures to evaluate exercise-mediated changes (increases, decreases) in low-grade inflammation and muscle damage [45]. In the present RCT trial, no detrimental effects on disease activity or disease damage were observed [14]. The present 1-year follow-up data support the previous literature, confirming that physical training (incl. high-intensity resistance exercise) does not accelerate nor negatively affect disease activity or disease damage in patients with established myositis [15–20].

### Limitations

Some limitations should be mentioned for the present follow-up study. Firstly, due to the COVID-19 pandemic, it was not logistically possible to recruit the number of patients needed to achieve optimal statistical power (80%), for the initial study. Secondly, the present 1-year follow-up was outside the scope of the initial RCT study, and therefore all statistical analyses were planned and performed post-hoc. The observations from the current study would have been strengthened, if the 1-year follow-up and its statistical plan had been a part of the original RCT design. Thirdly, for ethical reasons, we chose to include a dissemination seminar as well as training sessions for the control group following the end of the intervention period. This is likely to have caused a delayed exercise effect observed in the control at the 1-year follow-up, potentially, masking some of the exercise-induced differences between the groups. Lastly, to evaluate physical activity level at the 1-year follow-up, participants were asked to fill out training diaries. However, only a single participant did so, and consequently, it was impossible to adjust for physical activity level at 1-year follow-up. This likely introduced significant confounding, obscuring the true exercise response from the initial 16-week high-intensity resistance intervention.

Several strengths of the current study should be noted. Firstly, the present study adds to the limited knowledge of long-term follow-up in exercise-based RCTs involving patients with myositis. Secondly, the study achieved a high level of completion in both the initial RCT and the current 1-year follow-up. Lastly, the involvement of the advisory board in discussions on relevance, feasibility and study design significantly contributed to the overall quality of the study.

## Conclusion

This study is the first to present long-term effectiveness of high-intensity resistance training in patients with myositis, offering insights into the sustained effects thereof. The original RCT found immediate improvements in self-reported quality of life, muscle endurance and muscle strength following 16 weeks of resistance training. At the 1-year follow-up, indications of sustained improvements in self-reported quality of life were observed. Additionally, the improvements in muscle endurance persisted at the 1-year follow-up. Despite a potential ceiling effect on muscle strength outcomes, sustained strength benefits were observed, with no increases in disease activity or damage across time points or groups. Collectively, our findings reinforce the potential of high-intensity resistance training as a safe and long-term effective non-pharmacological treatment option for patients with myositis, offering sustained benefits without exacerbating disease activity or damage.

## Electronic supplementary material

Below is the link to the electronic supplementary material.

Supplementary Material 1

Supplementary Material 2

Supplementary Material 3

Supplementary Material 4

Supplementary Material 5

## Data Availability

Following the principles of transparency and reproducibility, we are committed to sharing the data that support the findings of this study. The datasets are available from the corresponding author upon reasonable request. Data will be provided under ethical guidelines and legal requirements, ensuring that participant confidentiality and privacy are protected.

## Declarations

### Disclaimer

No part of this manuscript, in its entirety, has been published elsewhere prior to this publication. It should be noted that both the baseline article (Jensen et al., 2024 [27]) and the RCT/intervention article (Jensen et al., 2024 [14]) include part of the demographics table, as it concerns the same cohort.

### Congress abstracts

Jensen KY, Aagaard P, Suetta C, et al. OP0065 effects of high-intensity resistance training for patients with myositis: 1-year follow-up on a randomised controlled intervention trial Annals of the rheumatic diseases 2024;83:95–96.
