# Supplementary material for: High-intensity resistance training in patients with myositis – 1-year follow-up on a randomised controlled trial
Source: Rheumatol Int. 2025 Apr 16;45(5):104. doi: 10.1007/s00296-025-05858-8 (PMC12003461; doi:10.1007/s00296-025-05858-8)
Supplement: Supplementary file 1 — Supplementary Material 1 [file 296_2025_5858_MOESM1_ESM.docx]

Table B – Post hoc analysis with “fat percentage“ as covariate – Differences in quality of life, functional capacity and muscle power at 1-year follow-up

|  | **Intervention (N = 15)** | | | | |  | **Control (N = 17)** | | | | | |  | | **Between-group difference** | | |
| --- | --- | --- | --- | --- | --- | --- | --- | --- | --- | --- | --- | --- | --- | --- | --- | --- | --- |
|  | Baseline to 1 year | |  | Post to 1 year | |  | Baseline to 1 year | |  | Post to 1 year | |  | | 1-year follow-up | | |  |
|  | *Difference*  *(95% CI)* | *P-value* |  | *Difference*  *(95% CI)* | *P-value* |  | *Difference*  *(95% CI)* | *P-value* |  | *Difference*  *(95% CI)* | *P-value* |  | | *Difference*  *(95% CI)* | | *P-value* |  |
| QoL – PCS  summary score | 5.7 (1.8; 9.6) | <0.01 |  | 0.0 (-4.1; 4.1) | 0.99 |  | -0.4 (-4.2; 3.3) | 0.83 |  | -0.6 (-4.6; 3.3) | 0.75 |  | | 6.1 (1.0; 11.3) | | 0.02 |  |
| QoL – MCS  summary score | 0.2 (-7.3; 7.7) | 0.96 |  | -5.3 (-13.6; 2.9) | 0.20 |  | 5.7 (-1.6; 13.0) | 0.12 |  | 3.0 (-4.9; 10.9) | 0.45 |  | | -5.5 (-15.1; 4.1) | | 0.26 |  |
| FI3  (%) | 10.2 (4.1; 16.3) | 0.02 |  | -6.5 (-12.7; -0.3) | 0.04 |  | -0.5 (-6.4; 5.4) | 0.87 |  | -6.1 (-12.1; -0.2) | 0.04 |  | | 10.7 (2.2; 19.1) | | 0.01 |  |
| 30-s STS  (reps) | 3.5 (1.9; 5.0) | <0.01 |  | 2.1 (0.5; 3.8) | 0.10 |  | 3.4 (1.9; 5.0) | <0.01 |  | 3.5 (2.0; 5.1) | <0.01 |  | | 0.0 (-2.1; 2.2) | | 0.99 |  |
| TUG  (s) | -0.8 (-1.2; -0.3) | <0.01 |  | 0.0 (-0.5; 0.5) | 0.95 |  | -0.4 (-0.9; 0.1) | 0.08 |  | -0.2 (-0.7; 0.3) | 0.39 |  | | -0.3 (-1.0; 0.3) | | 0.32 |  |
| 2MWT  (m) | 8.0 (1.1; 15.0) | 0.02 |  | -1.1 (-8.1; 5.9) | 0.75 |  | 10.6 (3.9; 17.2) | <0.01 |  | 2.3 (-4.4; 9.0) | 0.49 |  | | -2.5 (-12.1; 7.0) | | 0.60 |  |
| Balance  (s) | 0.6 (-0.2; 1.5) | 0.14 |  | 0.2 (-0.7; 1.1) | 0.67 |  | 0.8 (-0.1; 1.6) | 0.07 |  | 0.3 (-0.6; 1.2) | 0.46 |  | | -0.1 (-1.2; 0.9) | | 0.82 |  |
| Handgrip  (kg) | 2.8 (-0.2; 5.8) | 0.07 |  | 1.3 (-1.7; 4.3) | 0.40 |  | -0.9 (-3.8; 2.0) | 0.52 |  | -2.6 (-5.5; 0.3) | 0.08 |  | | 3.7 (-0.4; 7.9) | | 0.08 |  |
| LEP  (watts/kg) | 0.3 (0.0; 0.5) | 0.07 |  | 0.0 (-0.3; 0.3) | 0.96 |  | 0.1 (-0.1; 0.4) | 0.26 |  | 0.0 (-0.3; 0.3) | 0.93 |  | | 0.1 (-0.3; 0.5) | | 0.59 |  |
