# Supplementary material for: High-intensity resistance training in patients with myositis – 1-year follow-up on a randomised controlled trial
Source: Rheumatol Int. 2025 Apr 16;45(5):104. doi: 10.1007/s00296-025-05858-8 (PMC12003461; doi:10.1007/s00296-025-05858-8)
Supplement: Supplementary file 2 — Supplementary Material 2 [file 296_2025_5858_MOESM2_ESM.docx]

Table C – Post hoc analysis with “fat percentage“ as covariate – Differences in disease activity and disease damage at 1-year follow-up

|  | **Intervention (N = 15)** | | | | |  | **Control (N = 17)** | | | | | |  | | **Between-group difference** | | |
| --- | --- | --- | --- | --- | --- | --- | --- | --- | --- | --- | --- | --- | --- | --- | --- | --- | --- |
|  | Baseline to 1 year | |  | Post to 1 year | |  | Baseline to 1 year | |  | Post to 1 year | |  | | Post to 1 year | | |  |
|  | *Difference*  *(95% CI)* | *P-value* |  | *Difference*  *(95% CI)* | *P-value* |  | *Difference*  *(95% CI)* | *P-value* |  | *Difference*  *(95% CI)* | *P-value* |  | | *Difference*  *(95% CI)* | | *P-value* |  |
| PhGA  (0-100) | *2.8 (-0.7; 6.4)* | *0.11* |  | *2.5 (-1.2; 6.4)* | *0.18* |  | 3.2 (-0.2; 6.6) | 0.07 |  | 2.6 (-1.0; 6.1) | 0.15 |  | | *-0.3 (-5.0; 4.3)* | | 0.89 |  |
| PtGA  (0-100) | -4.1 (-7.7; -0.6) | 0.02 |  | -3.1 (-6.8; 0.6) | 0.10 |  | -1.7 (-5.1; 1.7) | 0.33 |  | -1.6 (-5.1; 2.0) | 0.37 |  | | -2.4 (7.2; 2.3) | | 0.31 |  |
| EMGA (0-100) | -1.0 (-3.6; 1.5) | 0.42 |  | -1.2 (-3.9; 1.5) | 0.37 |  | 0.9 (-1.5; 3.4) | 0.45 |  | 0.6 (-1.9; 3.2) | 0.63 |  | | -2.0 (-5.4; 1.5) | | 0.26 |  |
| MMT8  (0-80) | 1.9 (0.9; 3.0) | <0.01 |  | 0.0 (-1.0; 1.1) | 0.93 |  | 1.1 (0.1; 2.1) | 0.03 |  | 0.9 (-0.1; 2.0) | 0.07 |  | | 0.8 (-0.6; 2.2) | | 0.28 |  |
| HAQ (0-3) | 0.0 (-0.2; 0.1) | 0.57 |  | 0.1 (-0.1; 0.2) | 0.33 |  | 0.0 (-0.2; 0.1) | 0.47 |  | 0.0 (-0.1; 0.1) | 0.90 |  | | 0.0 (-0.2; 0.2) | | 0.93 |  |
| CK  (mmol/L) | -56 (-221; 110) | 0.51 |  | 8 (-183; 198) | 0.94 |  | -97 (-258; 65) | 0.24 |  | -110 (-292; 72) | 0.23 |  | | 41 (-159; 242) | | 0.68 |  |
| PhGD (0-100) | -6.0 (-12.3; 0.3) | 0.06 |  | 3.7 (-3.0; 10.4) | 0.27 |  | -5.0 (-11.1) | 0.10 |  | -0.5 (-6.9; 5.9) | 0.89 |  | | -1.0 (-9.4; 7.3) | | 0.81 |  |
| PtGD (0-100) | -5.4 (-8.9; -1.9) | <0.01 |  | -1.0 (-4.6; 2.5) | 0.56 |  | -4.2 (-7.6; -0.9) | 0.01 |  | -3.0 (-6.4; 0.4) | 0.08 |  | | -1.2 (-5.9; 3.6) | | 0.62 |  |
