# Supplementary material for: High-intensity resistance training in patients with myositis – 1-year follow-up on a randomised controlled trial
Source: Rheumatol Int. 2025 Apr 16;45(5):104. doi: 10.1007/s00296-025-05858-8 (PMC12003461; doi:10.1007/s00296-025-05858-8)
Supplement: Supplementary file 3 — Supplementary Material 3 [file 296_2025_5858_MOESM3_ESM.docx]

Table C – Post hoc analysis with “fat percentage“ as covariate – Differences in physical activity at 1-year follow-up

|  | **Intervention (N = 15)** | | | | | |  | | **Control (N = 17)** | | | | |  | | **Between-group difference** | | |  |
| --- | --- | --- | --- | --- | --- | --- | --- | --- | --- | --- | --- | --- | --- | --- | --- | --- | --- | --- | --- |
|  | Baseline to 1 year | |  | Post to 1 year | |  | | Baseline to 1 year | | |  | Post to 1 year | | |  | | Post to 1 year | | |
|  | *Difference*  *(95% CI)* | *P-value* |  | *Difference*  *(95% CI)* | *P-value* |  | | *Difference*  *(95% CI)* | | *P-value* |  | *Difference*  *(95% CI)* | *P-value* | |  | | *Difference*  *(95% CI)* | *P-value* | |
| IPAQ (Active hours/week) | -5.7 (-16.1; 4.8) | 0.28 |  | -4.0 (-14.9; 6.8) | 0.46 |  | | 4.1  (-5.9; 14.2) | | 0.41 |  | 7.0 (-3.4; 17.4) | 0.18 | |  | | -9.8 (-23.8; 4.2) | 0.17 | |
| IPAQ (Sitting hours/week) | 8.6 (-4.6; 21.8) | 0.20 |  | -4.9 (-19.3; 9.4) | 0.49 |  | | 6.8 (-6.0; 19.6) | | 0.29 |  | 4.0 (-9.7; 17.7) | 0.56 | |  | | 1.8 (-15.3; 18.9) | 0.84 | |
