# Supplementary material for: High-intensity resistance training in patients with myositis – 1-year follow-up on a randomised controlled trial
Source: Rheumatol Int. 2025 Apr 16;45(5):104. doi: 10.1007/s00296-025-05858-8 (PMC12003461; doi:10.1007/s00296-025-05858-8)
Supplement: Supplementary file 4 — Supplementary Material 4 [file 296_2025_5858_MOESM4_ESM.docx]

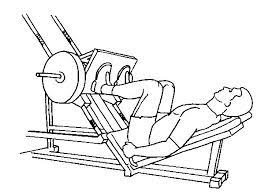
Træning i center

**Opvarmning**

- 10-15 minutter på kondicykel
- Alternativ: 10-15 minutters rask gang/lunte på løbebånd

Billede 1 - Ben pres

-
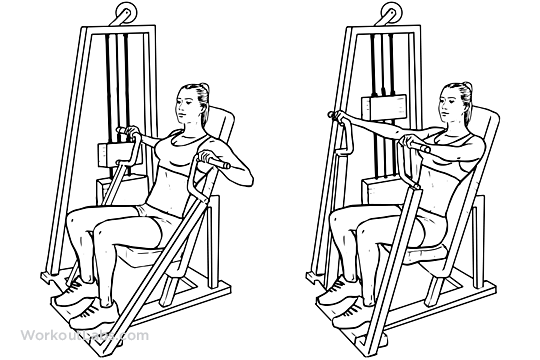
Alternativ: 5-10 minutter i romaskine (sat på laveste modstand (1))

**Styrketræningen** (alle øvelser laves i 3 sæt af 10 gentagelser, med 1-1,5 minutters pause)

- 1. øvelse (Arme)
  - Biceps curls med håndvægte - stående eller siddende
- 2. øvelse (Ben)
  - Ben pres i maskine (billede 1)

Billede 2 - Bænkpres

-
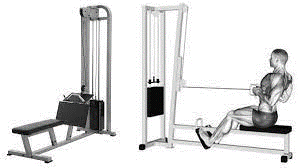
3. øvelse (Bryst)
  - Bænkpres i maskine (billede 2)
- 4. øvelse (Mave)
  - Almindelige og skrå mavebøjninger på måtte (10 af hver per sæt)
    - Alternativ: planke på måtte
- 5. øvelse (Ryg)
  - Kabeltræk i maskine (billede 3)
- 6. øvelse (ben)

Billede 3 - Kabeltræk

- -
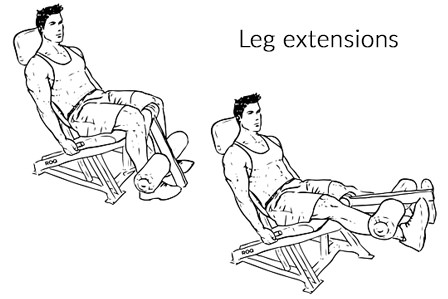
Knæekstension i maskine (billede 4)
    - Alternativ: Hamstring curls i maskine (billede 5)
- 7. øvelse (Skuldre)
  - Skulderpres (billede 6)
    - Håndvægt eller vægtstang
- Nedvarmning
  - 5 minutters stille og rolig gang på løbebånd
  - Alternativ: 10 minutter på cykel - stille og roligt

Billede 4 - Knæekstension

-
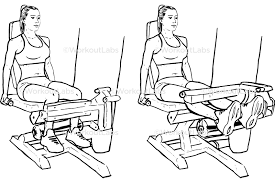
 Udstræk
  - 5-10 minutter med fokus på områder, der er relevant for dig
    - Kan være nakke, hofte, benmuskler eller fodled

**Trænings fremgang** (op i vægt)

-
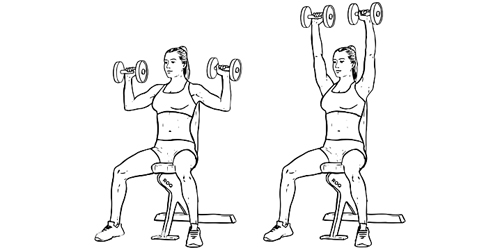
Hvis man efter 3. sæt af en øvelse kan tage yderligere 3 gentagelser overbevisende,
  bør man øge vægten af den øvelse

Billede 5 - Hamstring curls

- OBS - Hvis der er et stort spring til næste vægt, så start med kun at sætte vægten
  op i 1 sæt og arbejd dig op til den nye vægt i alle 3 sæt
- OBS – Gå ikke op i vægt i samme øvelse 2 uger i streg. Det minimerer risikoen
  for skader

Billede 6 - Skulderpres

Billede 6 – skulderpres


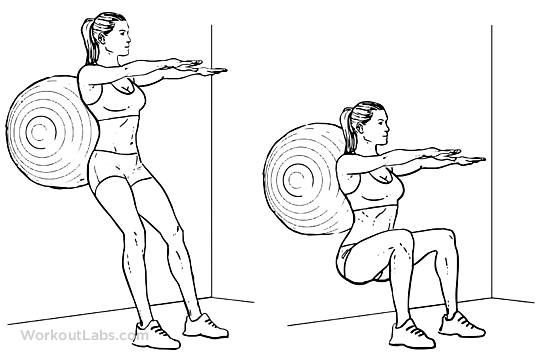
Hjemmetræning

**Opvarmning**

- 10-15 minutter på kondicykel
- Alternativ: 10-15 minutters rask gåtur
- Alternativ: 10 minutters høje knæløft på stedet og rask gang frem og tilbage
  i stuen (1 minuts knæløft, derefter 1 minuts gang, 1 minuts knæløft osv.)

Billede 1 - Air squat


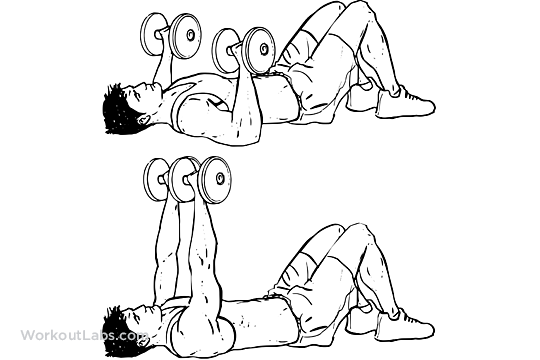


**Styrketræning** (alle øvelser laves i 3 sæt af 10 gentagelser, med 1-1,5 minutters pause)

- 1. øvelse (Arme)
  - Biceps curls med håndvægte - stående eller siddende

Billede 2 - Bænkpres

- - -
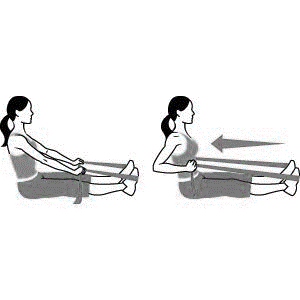
Alternativt: Biceps curls med elastik
- 2. øvelse (Ben)
  - Air squat – For god holdning, brug eventuelt en bold, som sættes
    i spænd imellem ryggen og en væg (billede 1)
    - Vægt kan tilføjes med rygsæk eller håndvægte holdt i hænderne
- 3. øvelse (Bryst)
  - Bænkpres med håndvægte liggende på gulvet/måtte (billede 2)
    - Alternativ: stående eller som siddende med elastik
      bundet til noget der står fast

Billede 3 - Kabeltræk

-
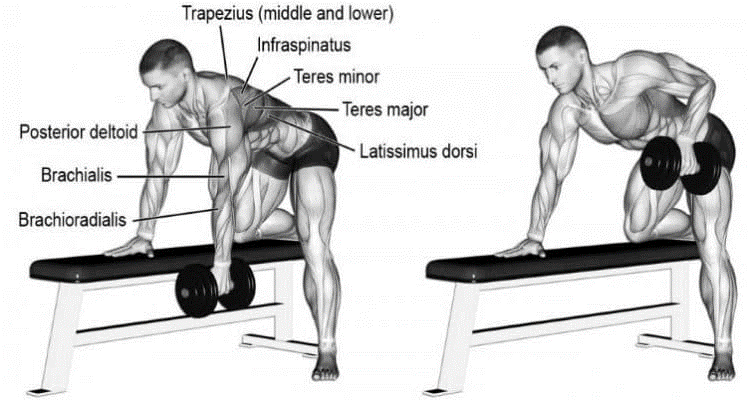
4. øvelse (Mave)
  - Almindelige og skrå mavebøjninger på måtte
    - Alternativ: Planke på måtte
- 5. øvelse (Ryg)
  - Kabeltræk med elastik – kan udføres enten en- eller to-arms (billede 3)
    - Alternativt: rygøvelse med håndvægt. Man står foroverbøjet med en arm
      og et ben på en stol, imens man med den løfter en håndvægt (billede 4)
- 6. øvelse (Ben)
  - Lunges (billede 5)
    - Vægt kan tilføjes med rygsæk eller håndvægte i hænderne

Billede 4 – Alternativ rygøvelse

-
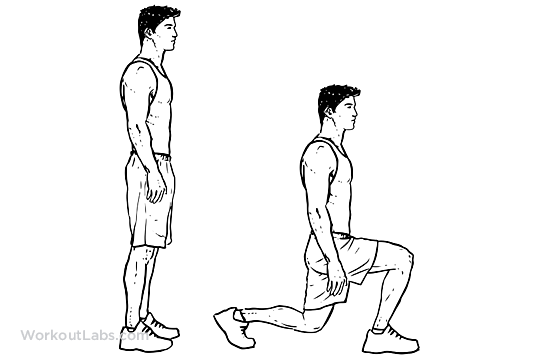
7. øvelse (Skuldre)
  - Skulderpres med håndvægte (billede 6)
    - Alternativ: skulderpres med elastik. Man sidder på en stol med
      elastikken under stolesædet og trækker derefter op i elastikken.
- Nedvarmning
  - 5 minutters stille og rolig gang
  - Alternativ: 10 minutter på cykel - stille og roligt
- Udstrækning
  - 5-10 minutter med fokus på områder, der er relevant for dig

Billede 5 - Lunges

- - -
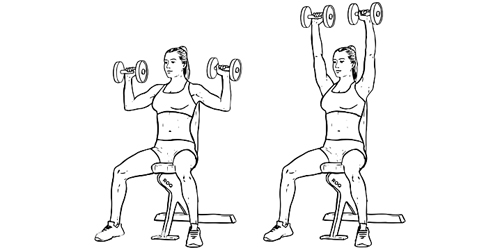
Kan være nakke, hofte, benmuskler eller fodled

**Trænings fremgang** (op i vægt)

- Hvis man efter 3. sæt af en øvelse kan tage yderligere 3 gentagelser overbevisende,
  så bør man øge vægten af den øvelse.
- OBS - Hvis der er et stort spring til næste vægt, så start med kun at sætte vægten
  op i 1 sæt og arbejd dig op til den nye vægt i alle 3 sæt.
- OBS – Gå ikke op i vægt i samme øvelse 2 uger i streg. Det minimerer risikoen for skader

Billede 6 - Skulderpres
