# Supplementary material for: High-intensity resistance training in patients with myositis – 1-year follow-up on a randomised controlled trial
Source: Rheumatol Int. 2025 Apr 16;45(5):104. doi: 10.1007/s00296-025-05858-8 (PMC12003461; doi:10.1007/s00296-025-05858-8)
Supplement: Supplementary file 5 — Supplementary Material 5 [file 296_2025_5858_MOESM5_ESM.pdf]

Træningsdagbog - uge

Navn	Kalender uge

Ugens træninger (sæt kryds)

	Mandag	Tirsdag	Onsdag	Torsdag	Fredag	Lørdag	Søndag
Hjemme	☐	☐	☐	☐	☐	☐	☐
Center	☐	☐	☐	☐	☐	☐	☐

Gennemsnitlig intensitet af ugens træninger

6	7	8	9	10	11	12	13	14	15	16	17	18	19	20

Meget let — Meget hårdt

Træthed i løbet af ugen (sæt en streg på linjen)

Ingen træthed — Ekstrem træthed

Smerter i løbet af ugen (sæt en streg på linjen)

Ingen smerter — Ekstreme smerter
